# C1q/TNF‐related peptide 8 (CTRP8) promotes temozolomide resistance in human glioblastoma

**DOI:** 10.1002/1878-0261.12349

**Published:** 2018-08-02

**Authors:** Thatchawan Thanasupawat, Aleksandra Glogowska, Maxwell Burg, Jerry Krcek, Jason Beiko, Marshall Pitz, Guo‐Jun Zhang, Sabine Hombach‐Klonisch, Thomas Klonisch

**Affiliations:** ^1^ Department of Human Anatomy and Cell Science Faculty of Medicine University of Manitoba Winnipeg Canada; ^2^ Department of Surgery Faculty of Medicine University of Manitoba Winnipeg Canada; ^3^ Department of Internal Medicine Faculty of Medicine University of Manitoba Winnipeg Canada; ^4^ Research Institute in Oncology and Hematology (RIOH) CancerCare Manitoba Winnipeg Canada; ^5^ ChangJiang Scholar's Laboratory Shantou University Medical College China; ^6^ Department of Medical Microbiology & Infectious Diseases Faculty of Medicine University of Manitoba Winnipeg Canada

**Keywords:** alkylating drug, base excision repair, CTRP8, DNA damage repair, MPG, RXFP1, temozolomide

## Abstract

The C1q/TNF‐related peptide 8 (CTRP8) has recently emerged as a novel ligand of the G protein‐coupled receptor RXFP1 in the fatal brain tumor glioblastoma (GBM). We previously demonstrated that the CTRP8‐RXFP1 ligand–receptor system promotes motility and matrix invasion of patient GBM and U87 MG cells by specific phosphorylation of PI3 kinase and protein kinase C. Here, we demonstrate a novel role for CTRP8 in protecting human GBM cells against the DNA alkylating damage of temozolomide (TMZ), the standard chemotherapy drug used to treat GBM. This DNA protective role of CTRP8 required a functional RXFP1‐STAT3 signaling cascade in GBM cells. We identified N‐methylpurine DNA glycosylase (MPG), a monofunctional glycosylase that initiates base excision repair pathway by generating an apurinic/apyrimidinic (AP) site, as a new CTRP8‐RXFP1‐STAT3 target in GBM. Upon TMZ exposure, treatment with CTRP8 reduced the formation of AP sites and double‐strand DNA breaks in GBM cells. This CTRP8 effect was independent of cellular MGMT levels and was associated with decreased caspase 3/7 activity and increased survival of human GBM. CTRP8‐induced RXFP1 activation caused an increase in cellular protein levels of the anti‐apoptotic Bcl members and STAT3 targets Bcl‐2 and Bcl‐XL in human GBM. Collectively, our results demonstrate a novel multipronged and clinically relevant mechanism by which the CTRP8‐RXFP1 ligand–receptor system exerts a DNA protective function against TMZ chemotherapeutic stress in GBM. This CTRP8‐RXFP1‐STAT3 axis is a novel determinant of TMZ responsiveness/chemoresistance and an emerging new drug target for improved treatment of human GBM.

**Abbreviations**
AP site: apurinic/apyrimidinic site
APE1: AP endonuclease 1
ARP: aldehyde reactive probe
ATM: ataxia telangiectasia mutated kinase
Bcl‐2: B‐cell lymphoma 2
Bcl‐XL: B‐cell lymphoma‐extra large
BER: base excision repair
cAMP: cyclic 3′–5′ adenosine monophosphate
CI: cell index
CTRP8: C1q/tumor necrosis factor‐related peptide 8
dI: deoxyinosine
DNA pol β: DNA polymerase β
DNA: deoxyribonucleic acid
GBM: glioblastoma
KD: knockdown
MGMT: *O* ^6^‐methylguanine‐DNA methyltransferase
MPG: N‐methylpurine DNA glycosylase
MTIC: 3‐methyl‐(triazen‐1‐yl) imidazole‐4‐carboxamide
OTM: olive tail moments
PCR: polymerase chain reaction
PI3K: phosphatidyl inositol 3 kinase
PKC: protein kinase C
RLN2: relaxin‐2
RTCA: real‐time cell analysis
RXFP1: relaxin family peptide receptor 1
siRNA: small interfering RNA
STAT3: signal transducer and activator of transcription 3
TMZ: temozolomide
XRCC1: X‐ray repair cross‐complementing group 1
γH2AX: phosphorylated histone 2A (Ser 139)

## 1 Introduction

The relaxin family peptide receptor 1 (RXFP1) is a G protein‐coupled receptor and relaxin‐2 (RLN2) is a major ligand in human tissues of normal and neoplastic origin (Halls *et al*., 2015; Klonisch *et al*., 2007). Relaxin was shown to promote vasodilation, cardioprotection, antifibrotic wound healing, and angiogenesis (Brecht *et al*., 2011; Conrad and Shroff, 2011; Du *et al*., 2010). In various tumors, the RLN2‐RXFP1 has emerged as an important ligand–receptor system involved in controlling growth, migration/tissue invasion, angiogenesis, and metastasis (Klonisch *et al*., 2007). Contrary to other tumors, malignant brain tumors such as Grade III anaplastic astrocytoma and Grade IV glioblastoma (GBM) express RXFP1 but fail to express RLN2 (Glogowska *et al*., 2013). Instead, we recently identified secreted adiponectin paralog C1q/tumor necrosis factor‐related peptide 8 (CTRP8) as a novel RXFP1 agonist in human GBM (Glogowska *et al*., 2013; Peterson *et al*., 2009). Of all 16 currently known CTRP members, CTRP8 is the least studied, in part, due to the fact that CTRP8 is a pseudogene in mice (Peterson *et al*., 2009). CTRPs are emerging as important regulators in metabolism, immune responses, and cancer (Kishore *et al*., 2004; Schaffler and Buechler, 2012; Seldin *et al*., 2014; Thanasupawat *et al*., 2015). All CTRPs are composed of four distinct structural domains and can form homo‐ or heterotrimers and multimeric complexes. CTRP8 shares close phylogenetic and sequence conservation with CTRP1 and CTRP6, and their C‐terminal globular domains share high conformational similarity with complement component C1q and tumor necrosis factor (TNF) (Kishore *et al*., 2004; Shapiro and Scherer, 1998). Located at the N terminus of the C1q/TNF globular domain of CTRP8 is the putative RXFP1 interacting site ‘AYAAFSV’ (Shemesh *et al*., 2008). In human GBM cells, the CTRP8‐mediated autocrine/paracrine RXFP1 activation resulted in elevated intracellular cAMP levels, PI3 kinase pathway activation, and the phosphorylation of PKC isoforms (Glogowska *et al*., 2013). Like RLN2 in other neoplastic models, the interaction of CTRP8 with RXFP1 promoted GBM matrix invasion and coincided with increased production and secretion of lysosomal protease cathepsin‐B, a known prognostic marker of GBM (Glogowska *et al*., 2013).

Glioblastoma is the most frequent and most aggressive form of primary brain tumor of the astrocytic lineage with a patient survival time of only 15–17 months. Treatment consists of extensive surgical resection followed by radiation and chemotherapy (Krex *et al*., 2007). The drug of choice in the treatment of GBM is temozolomide (TMZ), an imidazole derivative and second‐generation alkylating prodrug which undergoes spontaneous hydrolysis to the active metabolite 3‐methyl‐(triazen‐1‐yl) imidazole‐4‐carboxamide (MTIC). Treatment with TMZ results in DNA base methylation. The methylation at the N^7^ position of guanine (N^7^‐MeG; 80–85%) and the N^3^ position of adenine (N^3^‐MeA; 8–18%) constitute the majority of TMZ‐induced DNA methylations repaired by the base excision repair (BER) pathway. BER is the predominant DNA repair system in mammalian cells and repairs small cytotoxic DNA base lesions resulting from oxidized, alkylated, or deaminated nucleotides (Kim and Wilson, 2012; Krokan and Bjoras, 2013). The remaining 5–10% of TMZ‐induced DNA‐methylated lesions occur as O^6^‐MeG which is the substrate for the enzyme *O* ^6^‐methylguanine‐DNA methyltransferase (MGMT) (Sarkaria *et al*., 2008). The TMZ‐induced purine base alkylations N^3^‐MeA and N^7^‐MeG are the substrates for the monofunctional glycosylase N‐methylpurine DNA glycosylase (MPG, also known as alkylpurine‐DNA‐N‐glycosylase [APNG] or 3‐alkyladenine DNA glycosylase [AAG]). MPG initiates the first step of BER by removing the methylated base to generate an apurinic/pyrimidinic (AP) abasic site. The glycosylic backbone of the AP site is then cleaved by an AP lyase, like AP endonuclease 1 (APE1). This generates a cytotoxic 5′‐deoxyribosyl phosphate (dRP) residue which is commonly removed by the dRP lyase activity of DNA polymerase β (Sobol *et al*., 2000). DNA polymerase β adds the complementary base and the X‐ray repair cross‐complementing group 1 (XRCC1)/DNA ligase III complex performs the phosphodiester bond formation to complete BER (Krokan and Bjoras, 2013). Inhibition of BER in MPG overexpressing human glioma sensitizes these cells to TMZ *in vitro* and *in vivo*, but this cytotoxic effect is diminished at higher cellular levels of the rate‐limiting BER enzyme DNA polymerase β (Kim and Wilson, 2012; Tang *et al*., 2011).

In the present study, we have identified a novel role of the CTRP8‐RXFP1 ligand–receptor system in promoting the repair of TMZ‐induced alkylating DNA base damage in GBM. CTRP8 activated a newly discovered RXFP1‐STAT3 signaling pathway which caused enhanced resistance to DNA alkylating stress and increased survival in GBM upon TMZ treatment. This CTRP8‐RXFP1‐STAT3 signaling cascade may serve as a new mediator of TMZ chemoresistance in human GBM.

## 2 Materials and methods

### 2.1 Isolation of patient GBM cells and cell culture

Human GBM tissues were obtained from GBM patients treated at the local Health Science Centre. The study was approved by the University and Pathology ethics boards (ethics approval # H2010:116). Human GBM cells isolated from two GBM patients (GBM‐1/2) and the human U87MG glioblastoma cell line (Allen *et al*., 2016) were cultured in DME/F12 containing 10% FBS at 37 °C in a humidified 5% CO_2_ atmosphere. The medium was changed to DME/F12 with 1% FBS 24 h prior to the treatments. U87MG cells had been authenticated prior to this study.

### 2.2 Chemicals and reagents

Temozolomide (TMZ) was purchased from Sigma (Oakville, ON, Canada) and used at 1.5 mm which caused cell damage 24 h of incubation, respectively. STAT3 inhibitor VI, S3I‐201, was from EMD Millipore (Billerica, MA, USA). Cells were preincubated with inhibitors at 25 μm each for 60 min prior to additional treatment.

### 2.3 Recombinant protein production

Recombinant human full‐size C‐terminally Flag‐tagged CTRP8 in pET28a vector was produced in *Escherichia coli*. Recombinant CTRP8 was purified by His‐Gravity kit (GE Healthcare, Mississauga, ON, Canada) according to manufacturer's protocol and dialyzed against Tris buffer (50 mm Tris/HCl, 150 mm NaCl, pH 7.4) before determining the concentration by NanoVue spectrophotometer (GE Healthcare). The purity of the recombinant CTRP8 was assessed with 15% SDS/PAGE following Coomassie staining and immunoblot for anti‐Flag detection.

### 2.4 RNA silencing and PCR

For knockdown (KD) of RXFP1 in patient GBM cells, 5 × 10^4^ cells in six‐well plates were transfected with two different RXFP1 siRNA at a concentration of 100 nm [RXFP1‐1: (5′→3′) sense CCGUUUACCUGAUAAACCUtt, antisence AGGUUUAUCAGGUAAACGGgt; siRXFP1‐2: (5′→3′) sense GGAAGUAAUAAGAUUGAAAtt, antisense UUUCAAUCUUAUUACUUCCta (Ambion, Ottawa, ON, Canada) using siLentFect lipid reagent (Bio‐Rad, Mississauga, ON, Canada)]. Total RNA was collected for the detection of RXFP1 expression levels using RT‐PCR and quantitative real‐time PCR (qPCR) with the following primers: RXFP1 forward AAAAGAGATGATCCTTGCCAAACG, reverse CCACCCAGATGAATGATGGAGC; MPG forward GGTCCTAGTCCGGGGACTTCC, reverse CTTGTCTGGGCAGGCCCTTGC; and GAPDH forward CATCACCATCTTCCAGGAGCG, reverse TGACCTTGCCCACAGCC TTG. The qPCR was performed with a QuantStudio^®^ 3 system (Applied Biosystems, Ottawa, ON, Canada). The comparative *C*_T_ (ΔΔ*C*_T_) method was used for data analysis using quantstudio ^®^ design & analysis software (Applied Biosystem, Ottawa, ON, Canada). Samples were normalized to the expression of GAPDH.

### 2.5 xCELLigence ^®^ real‐time cell analysis (RTCA)

We performed xCELLigence real‐time cell cytotoxicity assays (ACEA Biosciences, Inc., San Diego, CA, USA). Patient GBM cells and U87MG cells were cultured on E‐plates and treated with CTRP8, TMZ, and siRXFP1 as indicated. Changes in cellular impedance are represented as cell index (CI) and were recorded every 15 min for 24 h upon treatment using rtca software (ACEA Biosciences, Inc., San Diego, CA, USA).

### 2.6 Caspase 3/7 activity assay

Caspase 3/7 activity assay was performed using Caspase‐Glo 3/7 reagent (Promega, Madison, WI, USA) according to the manufacturer's instructions. Caspase‐Glo 3/7 reagent was added to the samples in 96‐well plates and incubated for 4 h at room temperature (RT). Plates were spun at 350 rpm prior to detecting luminescence signal with a luminometer (Wallac, PerkinElmer, Boston, MA, USA).

### 2.7 Single‐cell gel electrophoresis assay (alkaline comet assay)

GBM cells (5 × 10^4^ cells) plated in 6‐well plates were treated, and DNA damage was assessed using a Comet assay kit (Trevigen, Gaithersburg, MD, USA). Cells were embedded in low‐melting‐point agarose on glass slides. Once the agarose was solidified, slides were maintained in prechilled lysis solution at 4 °C for 45 min before being incubated in an alkaline solution for 20 min at RT followed by single‐cell gel electrophoresis with fresh electrophoresis buffer for 15 min at 25 V 0.8 amps. Slides were dehydrated with 70% and 100% ethanol for 20 min and stained with SYBR green. Comet images were acquired using a Z2 microscope (Zeiss, Jena, Germany). Comet olive tail moments (OTM; product of the tail length and the fraction of total DNA in the tail), a measure of DNA damage, were quantified for 50 cells per treatment using the comet assay iv software (Perceptive, Bury St Edmunds, UK).

### 2.8 Immunofluorescence

Immunofluorescence detection of γH2AX was described previously (Thanasupawat *et al*., 2017a). Briefly, patient GBM cells on cover slips were treated with siRNA for RXFP1 silencing 24 h prior to treatment with CTRP8 and/or TMZ. Cells were fixed with 3.7% formaldehyde for 20 min at RT. Nonspecific antibody binding sites were blocked for 2 h at RT with 1% BSA in 0.01% Triton X‐100 plus 5% rabbit normal serum (blocking buffer; Sigma). GBM cells were immunostained overnight with 1 : 5000 γH2AX (EMD; Millipore) in blocking buffer at 4 °C prior to incubation for 1 h at RT with AlexaFlour‐594‐conjugated rabbit anti‐mouse (Invitrogen, Thermo Scientific). For nuclear staining, cells were counterstained with 0.1 μg·mL^−1^ DAPI and mounted with Fluoromount aqueous mounting medium (both Sigma). Cells were imaged with a Z2 microscope and zen imaging software (Zeiss). Intensity quantification of immunofluorescence signal for γH2AX foci was analyzed using image j software (National Institutes of Health, Bethesda, MD, USA). A total of 100 nuclei per each treatment for all cell lines were analyzed. The results are represented as a graph with relative fluorescence intensity.

### 2.9 Western blot analysis

Proteins were separated on 10% and 12% SDS/PAGE gels and transferred to nitrocellulose membranes. For immunodetection, nonspecific protein binding sites were blocked by incubation with 5% nonfat milk in TBS/T for 1 h at RT. Primary antibodies [1 : 1000 of pSTAT3^Tyr705^, pSTAT3^Ser727^, total STAT3, γH2AX, XRCC1, MGMT, Bcl‐2, Bcl‐XL (all Cell Signaling Technologies, Boston, MA, USA), 1 : 2000 of APE1, 1 : 3000 of MPG, 1 : 500 of DNA Polβ (all Abcam, Toronto, ON, Canada), and 1 : 10 000 for β‐actin (Sigma)] were incubated at 4 °C overnight. Membranes were washed 3× in TBS/T for 5 min each at RT before adding HRP‐conjugated secondary antibodies for 1 h. Specific binding was visualized with ECL solution (Thermo Scientific). All western blots were performed using Bio‐Rad Laboratories Inc (Bio‐Rad, Mississauga, ON, Canada) system, including ChemiDoc MP gel documentation and image lab software for quantitate analysis of proteins signals. Percentage of relative intensity was display as graphs representing three independent experiments for each of the cell line use in the study.

### 2.10 N‐methylpurine DNA glycosylase (MPG) molecular beacon activity assay

The MPG activity assay was performed on U87MG cells as described previous (Svilar *et al*., 2012). Beacon oligodeoxyribonucleotides (MPG probe: 5′‐ 6‐FAM/GCACT/**X**/TTGAATT GACACGCCATGTCGATCAATTCAATAGTGC/3Dab/‐3′, control probe: 5′‐ 6‐FAM/GCACTATTGAATTGACACGCCATGTCGATCAATTCAATAGTGC/3Dab/‐3′; X is deoxyinosine) were purchased from Integrated DNA Technologies (Coralville, IA, USA). Stem loop formation of the beacons was confirmed by heating the oligonucleotides to 95 °C for 3 min followed by slow cooling overnight at RT. Upon hairpin loop formation, no fluorescence signal was emitted and the beacon remained stable at 37 °C. When the hairpin loop beacon was reheated to 95 °C, fluorophore and quencher separated as the oligonucleotides unfolded, resulting in maximum fluorescence signal. Nuclear protein lysates were extracted with NE‐PER nuclear/cytoplasmic extraction reagents (Thermo Scientific). Ten micrograms of nuclear protein lysates was incubated with 40 nm beacon probe, and fluorescence was detected at 37 °C every 20 s for 120 min using a QuantStudio^®^ 3 system.

### 2.11 Detection of AP sites in genomic DNA

U87MG cells were pretreated with 100 ng·mL^−1^ of CTRP8 in 1% FBS for 24 h prior to treatment with 1.5 mm TMZ for 15 min. Genomic DNA was extracted using Genomic DNA Mini Kit (Thermo Scientific) according to the manufacturer's instructions. The ARP labeling and quantification of AP sites were performed by AP sites assay kit (Dojindo Molecular Technologies, Burlington, ON, Canada). Ten microliters of genomic DNA (100 μg·mL^−1^) in TE buffer was incubated with 10 μL of 5 mm ARP solution at 37 °C for 1 h. ARP‐labeled DNA in DNA binding solution was added to a 96‐well plate and incubated at 37 °C overnight in the dark. Wells were washed 5 times with washing buffer, HRP–Streptavidin solution was added and incubated at 37 °C for 1 h. Wells were washed again 5 times and incubated with substrate solution at 37 °C for 1 h prior to absorbance was measured at 630 nm with Synergy H1 microplate reader (BioTek, Winooski, VT, USA). Standard ARP DNA kit solutions determined 1–40 AP sites per 100 000 bp, and data are presented as number of AP sites per 100 000 nucleotides. All treatments were performed in triplicate.

### 2.12 Statistical analysis

All experiments were carried out at least in triplicate. Results are showed as mean ± standard deviation (SD). Data were analyzed with graphpad prism 6 statistical software using two‐way ANOVA with post hoc Tukey's HSD (honestly significant difference). *P* values less than 0.05 was considered significant. The level of significance was defined as *P < 0.05, **P < 0.01, ***P < 0.001, and ****P < 0.0001.

## 3 Results

### 3.1 CTRP8 activates a novel RXFP1‐STAT3 signaling pathway in GBM cells

**Figure 1:**
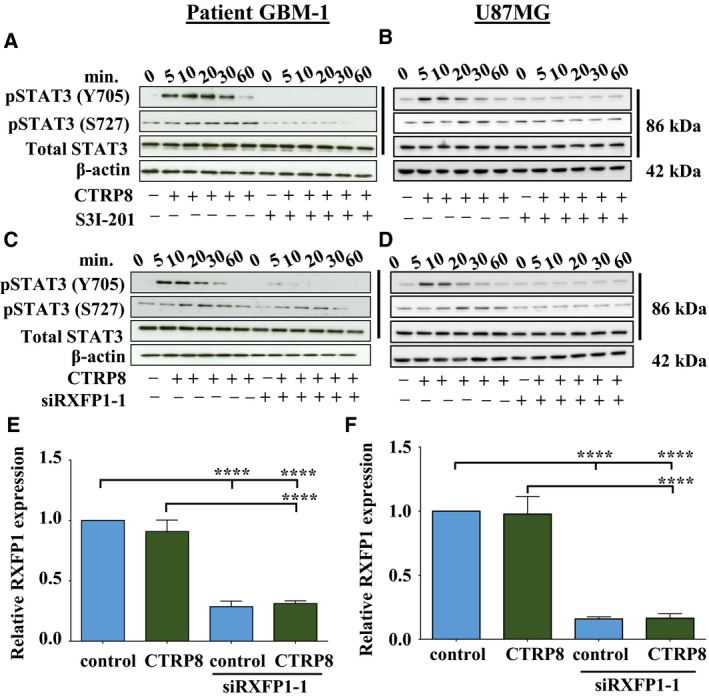
CTRP8 promotes STAT3 signaling in GBM. Exposure of human GBM‐1 with human recombinant CTRP8 (100 ng·mL^−1^) resulted in the phosphorylation of STAT3 at Tyr705 and Ser727 in patient GBM cells (A, C) and U87MG (B, D), whereas total STAT3 protein levels remained unchanged (A–D). Pretreatment with the specific STAT3 inhibitor S3I‐201 abolished the ability of CTRP8 to cause STAT3 phosphorylation in patient GBM‐1 cells (A) and U87MG (B). This CTRP8 effect was more pronounced for the pSTAT3^Y705^ than pSTAT3^S727^ residue. Similarly, siRXFP1 knockdown (KD; siRXFP1‐1) diminished phosphorylation of both pSTAT3^Y705/S727^ residues and abolished the ability of CTRP8 to induce STAT3 phosphorylation in patient GBM‐1 (C) and U87MG cells (D). β‐Actin served as loading control in all blots. Representative examples of qPCR results demonstrate the significant downregulation of RXFP1 transcripts upon siRXFP1‐1 treatment in patient GBM‐1 (E) and U87MG (F) cells. Quantitative analysis from three independent experiments (two‐way ANOVA; data are shown as mean ± SD; ****P < 0.0001) are shown.

Aberrant STAT3 signaling is a hallmark of gliomagenesis and has important therapeutic implications in GBM (Birner *et al*., 2010). Treatment of patient GBM‐1/2 (Fig. 1A, C, E; Fig. S1A–C) and U87MG (Fig. 1B, D, F) with CTRP8 resulted in STAT3 activation with pSTAT3^Y705^ phosphorylation as early as 5 min after treatment. A subtle phosphorylation was observed for STAT3^S727^ upon stimulation with CTRP8 in patient GBM‐1 (Fig. 1A, C; Fig. S1A, B) and U87MG (Fig. 1B, D). STAT3 inhibitor S3I‐201 effectively blocked STAT3 phosphorylation in CTRP8‐treated patient GBM‐1 (Fig. 1A; Fig. S1A) and U87MG (Fig. 1B) cells but had no effect on total STAT3 levels. CTRP8‐mediated STAT3 activation was critically dependent on the presence of RXFP1 in human GBM cells. Specific siRNA‐mediated RXFP1 KD in patient GBM‐1 and U87MG with two different siRXFP1‐1/2 constructs abolished the ability of CTRP8 to cause STAT3 phosphorylation in patient GBM (Fig. 1C; Fig. S1B) and U87MG (Fig. 1D). QPCR confirmed the successful siRXFP1 KD with siRXFP1/2 in patient GBM‐1 (Fig. 1E; Fig. S1C) and U87MG cells (Fig. 1F) and demonstrated that CTRP8 did not alter endogenous RXFP1 mRNA levels (Fig. 1E, F; Fig. S1C). Similar results were obtained in patient GBM‐2 cells treated with siRXFP1‐2, indicating that the effects detected with siRXFP1 treatment were likely not the result of siRNA‐mediated off‐target effects (Fig. S2A, B). Collectively, these results identified CTRP8 as a novel inducer of an RXFP1‐STAT3 signaling cascade in human GBM.

### 3.2 CTRP8 protects GBM cells against DNA damage by the alkylating drug temozolomide

**Figure 2:**
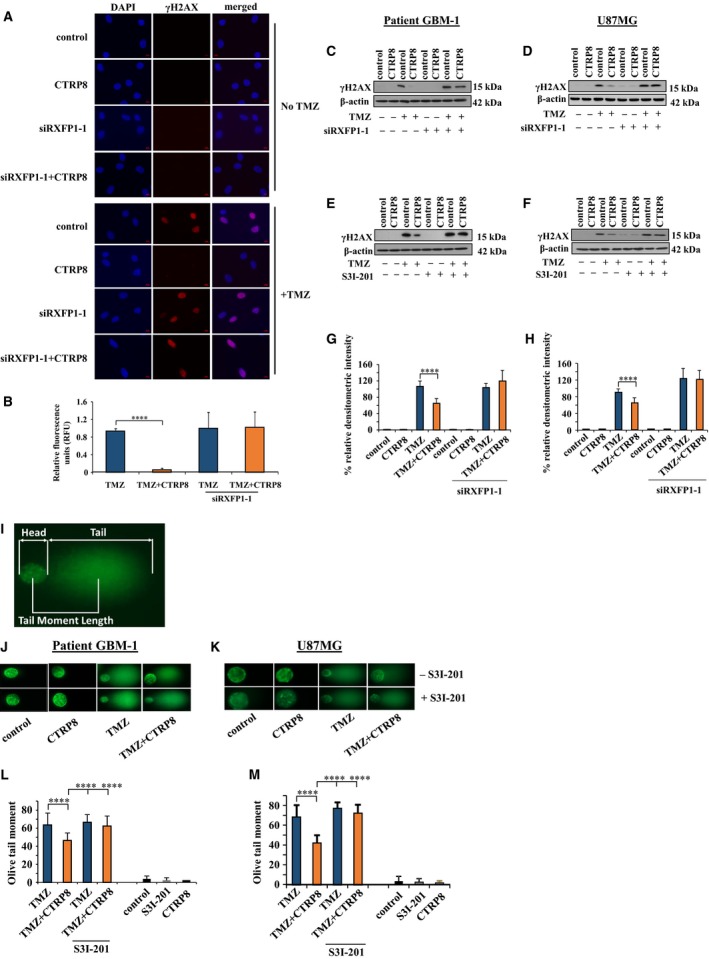
CTRP8 attenuates TMZ‐induced DNA damage. γH2AX, a marker of double‐strand (ds) DNA breaks, was detected by immunofluorescence in patient GBM (A). Treatment with TMZ (1.5 mm) resulted in a significant increase in γH2AX foci (red) in DAPI‐stained nuclei (blue) compared to medium controls (A). Pretreatment for 24 h with CTRP8 (100 ng·mL^−1^) caused a marked reduction in γH2AX foci upon exposure to TMZ compared to patient GBM‐1 cells treated with TMZ alone (A). This CTRP8‐mediated DNA protective effect was abolished upon siRXFP1‐1 KD (A). The results of the quantification of fluorescence intensity of γH2AX foci for 100 nuclei per treatment group are shown (B). Upon TMZ treatment, western blot analysis (C–F) revealed a marked reduction in γH2AX protein in the presence of CTRP8 in patient GBM‐1 (C, E) and U87MG (D, F). This DNA protective effect of CTRP8 was blocked upon siRXFP1‐1 treatment in patient GBM‐1 (C) and U87MG (D). Similar results were obtained with STAT3 inhibitor S3I‐201 in patient GBM‐1 (E) and U87MG (F). β‐Actin served as loading control. Representative western blot images are shown. Densitometry of western blot signal for γH2AX in patient GBM‐1 (G) and U87MG (H) was normalized to β‐actin. We employed Comet assay to quantify DNA fragmentation as determined by olive tail moment (OTM) in individual human GBM‐1 cells (I, J). Pretreatment for 24 h with CTRP8 (100 ng·mL^−1^) prior to TMZ exposure (1.5 mm) resulted in decreased OTM, as shown in representative agarose gel images of comets from patient GBM‐1 cells (I, J). The OTM was determined as an index of both comet tail length and the amount of DNA in tail as quantified by SYBR green fluorescence dye (L). Quantitative analysis of OTM from 50 cells for each treatment group revealed that CTRP8 caused a marked reduction in dsDNA breaks. This protective CTRP8 function was lost upon treatment with S3I‐201 in patient GBM‐1 (K) and U87MG (M). CTRP8 and S3I‐201 alone failed to cause dsDNA breaks and resulted in negligible OTM (K, M). Quantitative analysis from three independent experiments (two‐way ANOVA; data are shown as mean ± SD; ****P < 0.0001) are shown.

The STAT3 signaling pathway is associated with TMZ chemoresistance in GBM, but the underlying mechanisms are unclear (Villalva *et al*., 2011). Here, we show that RXFP1 agonist CTRP8 (Glogowska *et al*., 2013) mitigated the ability of first‐line GBM drug TMZ to induce DNA damage. Patient GBM‐1/2 cells (Fig. 2A, B; Fig. S3A, B) and U87MG (Fig. S3D, E) exposed to TMZ demonstrated strong immunofluorescence for nuclear γH2AX, an established marker for double‐strand (ds) DNA breaks. However, GBM‐1/2 cells cotreated with TMZ and CTRP8 showed markedly reduced nuclear γH2AX fluorescence, while CTRP8 alone did not elicit dsDNA breaks in patient GBM (Fig. 2A, B; Fig. S3A, B) or U87MG (Fig. S3D, E). The CTRP8 protective effect against dsDNA damage resulting from unrepaired TMZ‐induced DNA lesions was RXFP1‐dependent and abolished by siRXFP1 KD in patient GBM‐1/2 (Fig. 2A, B; Fig. S3A, B) and U87MG (Fig. S3D, E). Corresponding IgG control experiments failed to show specific immunofluorescence as shown for patient GBM‐2 (Fig. S3C, F) and U87MG (Fig. S3F).

Quantitative western blot analysis revealed that treatment with CTRP8 of patient GBM‐1/2 (Fig. 2C, E, G; Fig. S4A–C) and U87MG (Fig. 2D, F, H) resulted in a marked reduction in phosphorylated γH2AX protein upon TMZ treatment as compared to TMZ treatment alone. The presence of RXFP1 was critical for CTRP8 to elicit its DNA protective effect in the presence of TMZ and was lost upon siRXFP1‐1 KD in GBM‐1/2 (Fig. 2C, G; Fig. S4A, C) and U87MG (Fig. 2D, H). Similar results were obtained in patient GBM‐2 cells upon treatment with a siRXFP1‐2 (Fig. S2C). In addition, STAT3 inhibitor S3I‐201 blocked the ability of CTRP8 to attenuate γH2AX protein levels upon TMZ treatment in patient GBM‐1/2 (Fig. 2E; Fig. S4B) and U87MG (Fig. 2F).

We assessed the extent of CTRP8‐mediated protection against DNA damage induced by TMZ at the single cell level (Fig. 2I–M; Fig. S4D, E). Comet assay permits the quantification of dsDNA fragmentation in the nucleus at the level of a single GBM cell (Alapetite *et al*., 1999). Exposure to TMZ increased the olive tail moment (OTM) (Fig. 2I) in patient GBM‐1/2 (Fig. 2J, L; Fig. S4D, E) and U87MG (Fig. 2K, M). Quantitative analysis of the comets showed that the TMZ‐induced OTM was markedly reduced in the presence of CTRP8 in patient GBM‐1/2 (Fig. 2L; Fig. S4E) and U87MG (Fig. 2M). This DNA protective function of CTRP8 in the presence of TMZ was abrogated by the specific STAT3 inhibitor S3I‐201 in patient GBM‐1/2 (Fig. 2J, L; Fig. S4E) and U87MG (Fig. 2K, M), while CTRP8 or S3I‐201 alone had no effect (Fig. 2L, M; Fig. S4E). These data demonstrated a novel protective role of the CTRP8‐RXFP1‐STAT3 signaling pathway against TMZ chemotherapeutic stress in human GBM.

### 3.3 TMZ resistance induced by CTRP8 involves increased MPG and BER activity

**Figure 3:**
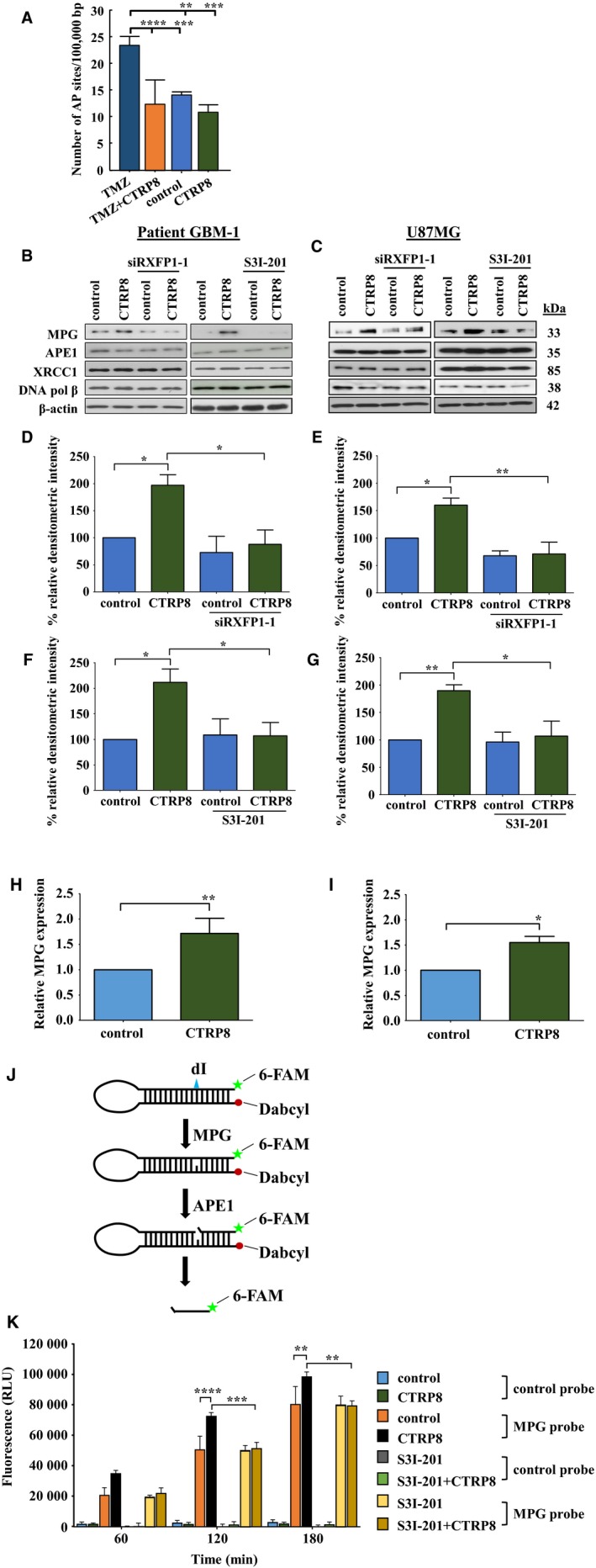
CTRP8 reduces the number of AP sites and enhances BER. We quantified AP sites in genomic DNA to determine the cause of TMZ‐induced DNA damage. The endogenous level of AP sites was about 12–15 sites/10^5^ bp in our patient GBM‐1 model (A). TMZ alone triggered a marked upregulation of AP sites in patient GBM‐1 cells (A). A significant reduction in the number of AP sites comparable to levels detected in untreated control cells was observed upon pretreatment of patient GBM‐1 cells with CTRP8 followed by TMZ exposure (A). CTRP8 treatment alone did not alter the number of AP sites (A). Western blot analysis of important BER proteins showed that CTRP8 (100 ng·mL^−1^) induced the specific and exclusive upregulation of N‐methylpurine DNA glycosylase (MPG) protein, a key factor in initiating BER, in patient GBM‐1 (B) and U87MG (C). Importantly, siRXFP1 KD and STAT3 inhibition abolished this CTRP8 mediated increase in MPG protein (B–G) as shown for patient GBM‐1 (B, D, F) and U87MG (C, E, G). The increase in MPG protein coincided with a significant upregulation of MPG transcripts upon CTRP8 treatment as revealed by QPCR analysis in patient GBM‐1 (H) and U87MG (I). We used an MPG‐specific molecular beacon activity assay on U87MG nuclear lysates which utilizes a specific MPG deoxyribonucleotide probe with a fluorophore (6‐FAM) attached at the 5′‐end and a quencher (3Dab) at the 3′‐end (Svilar *et al*., 2012) (J). This MPG oligo probe contains a deoxyinosine (dI) base as MPG recognition site. The exclusive MPG cleavage at this site releases a 6‐bp DNA fragment with attached 5′‐fluorophore which dissociates from its quencher to generate a fluorescence signal which is proportional to MPG activity and can be quantified by qPCR (J). An identical control oligo lacking the dI base and, thus, is not cleaved by MPG was used as control. Quantification of fluorescence intensities reflecting MPG activities measured at 60, 120, and 180 min is shown (K). Endogenous MPG exclusively cleaved the MPG probe, and CTRP8 treatment caused a further significant increase in MPG activity which was absent in the presence of S3I‐201 (K). Quantitative analysis from three independent experiments (two‐way ANOVA; data are shown as mean ± SD; *P < 0.05, **P < 0.01, ***P < 0.001, and ****P < 0.0001) are shown.

The removal of TMZ‐induced methylated DNA bases by the base excision repair (BER) pathway generates apurinic/apyrimidinic (AP) single‐stranded (ss) DNA sites which are fragile and can progress into dsDNA breaks (Krokan and Bjoras, 2013). We reasoned that a DNA protective role of CTRP8 may involve a reduction in the number of detectable AP sites in genomic DNA. Here, we show that TMZ significantly increased the number of AP sites per 10^5^ nucleotides from an average of 15 (control) to 24 AP sites (Fig. 3A). CTRP8 markedly reduced the number of AP sites in TMZ‐treated patient GBM‐1 cells by >40% to levels of untreated GBM cells (Fig. 3A). To determine a molecular mechanism that can account for this remarkable DNA protective effect of CTRP8, we assessed the amounts of key cellular BER proteins in the patient GBM‐1/2 models (Fig. 3B; Fig. S5A) and U87MG (Fig. 3C). Exposure to CTRP8 caused an exclusive increase in protein production of the monospecific DNA glycosylase N‐methylpurine DNA glycosylase (MPG), a key BER‐initiating enzyme which removes altered DNA bases and generates AP sites (Kim and Wilson, 2012; Krokan and Bjoras, 2013), whereas other BER members, including APE1, XRCC1, or DNA polymerase B (DNA pol β), remained unchanged as determined by quantitative western blot analysis in patient GBM‐1/2 (Fig. 3B, D, F; Fig. S5A–C) and U87MG (Fig. 3C, E, G). This increase in MPG protein coincided with the ability of CTRP8 to enhance MPG gene expression in patient GBM‐1/2 (Fig. 3H; Fig. S5D) and U87MG (Fig. 3I). The presence of a functional RXFP1 in GBM was required for increased MPG protein production as determined by siRXFP1 treatment in patient GBM‐1/2 (Fig. 3D; Fig. S5B) and U87MG (Fig. 3E). Similar results were obtained with a different siRNA (siRXFP1‐2) to suppress RXFP1 expression in patient GBM‐2 (Fig. S2D). Quantitative western blot data showed that the increase in MPG protein was also sensitive to STAT3 activation. STAT3 inhibitor S3I‐201 blocked this increase as shown in patient GBM‐1 (Fig. 3F) and U87MG (Fig. 3G).

Next, we employed a real‐time molecular beacon assay to specifically measure MPG activity and show that the observed increase in MPG protein content observed upon CTRP8 treatment translated into increased MPG enzymatic activity. The MPG molecular beacon assay uses a specific MPG deoxyribonucleotide probe with a fluorophore (6‐FAM) attached at the 5′‐end and a quencher (3Dab) at the 3′‐end (Svilar *et al*., 2012). The MPG molecular probe contains a deoxyinosine (dI) base that is specifically recognized and exclusively cleaved by the MPG enzyme to release a short DNA fragment and the attached 5′‐fluorophore from its quencher to generate a fluorescence signal which can then be quantified by qPCR (Fig. 3J). An identical molecular beacon lacking the MPG recognition site was used as a control. When nuclear lysates of U87MG were incubated with the MPG beacon, we observed a steady increase in fluorescence signal over time with the MPG probe in cell lysates of untreated U87MG, reflecting endogenous MPG activity (Fig. 3K). A further significant increase in MPG activity was observed upon exposure to CTRP8 which was reduced to endogenous MPG activity levels by STAT3 inhibitor SI3‐201 (Fig. 3K). The absence of fluorescence signals in cell lysates incubated with the control probe lacking the MPG cleavage site showed that the MPG beacon assay specifically detected MPG activity (Fig. 3K). These results demonstrated that CTRP8 can increase both MPG protein content and MPG activity in human GBM cells. The DNA protective role of CTRP8 was not attributable to changes in MGMT protein. Both MGMT and STAT3 have been shown to mediate TMZ resistance in glioblastoma (Hegi *et al*., 2005; Kohsaka *et al*., 2012). In the patient GBM cells studied, CTRP8 failed to alter MGMT protein levels and U87MG is devoid of MGMT (Fig. S6) (Thanasupawat *et al*., 2017b). Collectively, we identified the key BER monofunctional glycosylase MPG as a novel target and mediator of DNA protection of the CTRP8‐RXFP1‐STAT3 signaling cascade in human GBM cells.

### 3.4 CTRP8 promotes glioblastoma survival

**Figure 4:**
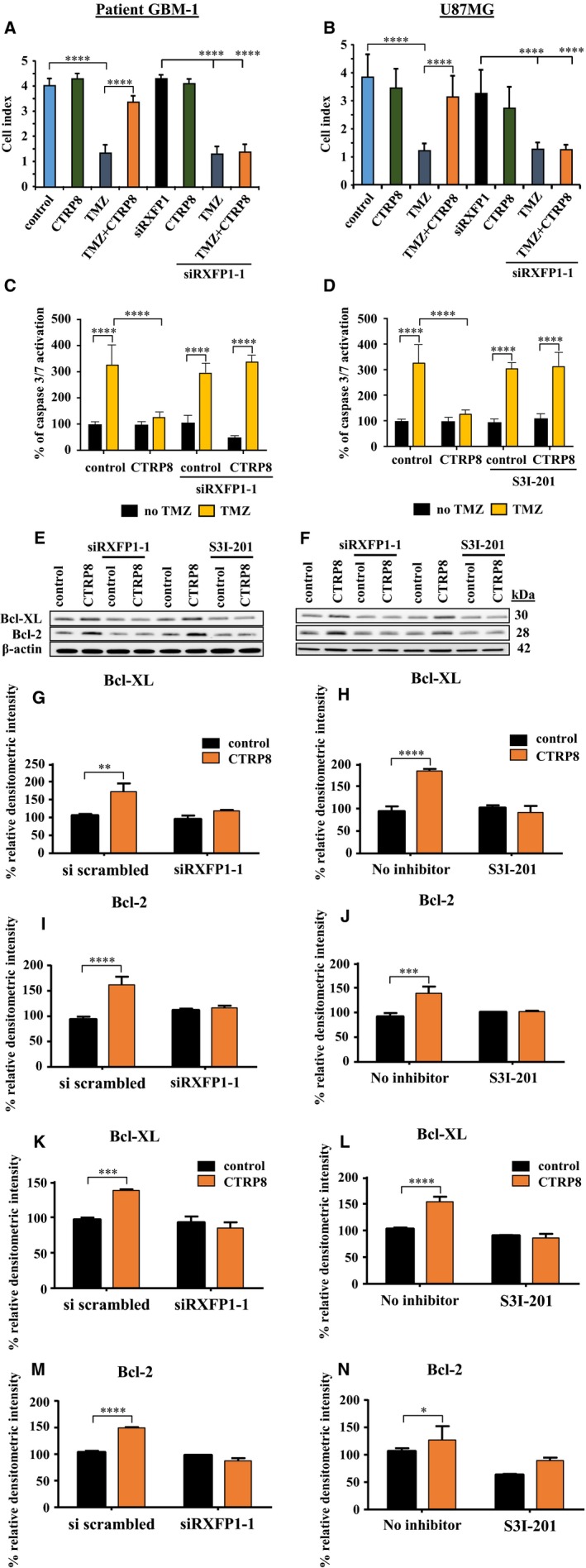
CTRP8 promotes GBM cell survival. Real‐time xCELLigence assays were used to quantify the effect of CTRP8 on TMZ‐induced cytotoxicity. Measurements were taken every 15 min, and cell indices collected at 24 h of incubation are shown (A, B). We observed a strong TMZ‐mediated cytotoxicity in patient GBM‐1 (A) and U87MG (B). Treatment with CTRP8 largely abolished the cytotoxic effect of TMZ in patient GBM‐1 (A) and U87MG (B). This CTRP8 protective function was dependent on the presence of functional RXFP1, as siRXFP1 KD abolished this effect, and treatment with CTRP8 and siRXFP1 alone had no effect in GBM‐1 (A, B). Caspase 3/7 activity assays (C, D) confirmed that CTRP8 protected patient GBM‐1 (C) and U87MG (D) against the cytotoxic effects of TMZ. This protective function CTRP8 was lost upon siRXFP1 KD (C) and STAT3 inhibition (D) as demonstrated in representative results for siRXFP1‐1 KD in patient GBM‐1 (C) and S3I‐201 treatment in U87MG (D). Western blot analysis revealed that CTRP8 treatment caused the RXFP1‐ and STAT3‐dependent upregulation of anti‐apoptotic Bcl members and STAT3 targets Bcl‐XL and Bcl‐2 (E, F). Densitometry results of western blots for Bcl‐XL are shown for patient GBM‐1 (G, H) and U87MG (K, L) and for Bcl‐2 with patient GBM‐1 (I, J) and U87MG (M, N). Quantitative analysis from three independent experiments (two‐way ANOVA; data are shown as mean ± SD; *P < 0.05, **P < 0.01, ***P < 0.001, and ****P < 0.0001) are shown.

**Figure 5:**
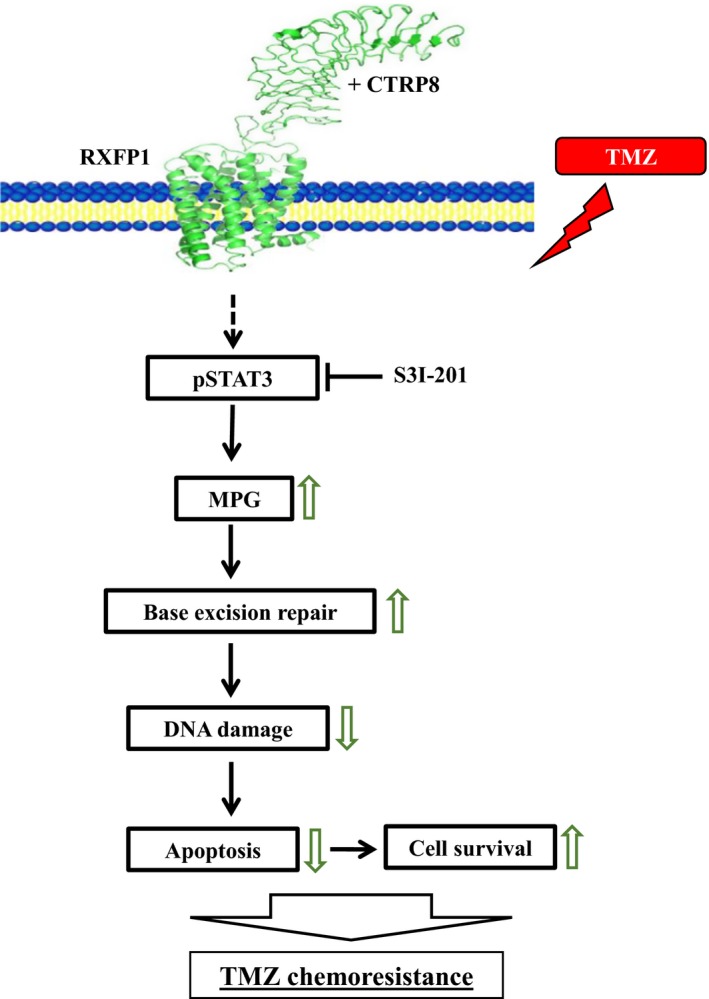
Schematic model of the CTRP8‐RXFP1‐STAT3 signaling axis in human GBM. We propose a model in which the interaction of CTRP8 with membrane‐anchored RXFP1 triggers a pSTAT3 signaling cascade in human GBM. STAT3 activation enhances MPG‐dependent BER, thereby reducing DNA damage and promoting GBM survival. The latter includes the upregulation of anti‐apoptotic STAT3 targets Bcl‐2 and Bcl‐XL. Collectively, this establishes the CTRP8‐RXFP1‐STAT3 cascade as a novel oncogenic signaling pathway that promotes TMZ chemoresistance in human high‐grade glioma.

We reasoned that the DNA protective function of the CTRP8‐RXFP1‐STAT3 signaling cascade would be most effective if CTRP8 also promoted cell survival mechanisms in glioblastoma. Real‐time cell analysis (RTCA) cytotoxicity assays revealed that TMZ treatment caused significant cell death, indicated by a marked decrease in cell index (CI) compared to untreated controls in patient GBM‐1/2 (Fig. 4A; Figs S2E and S5E) and U87MG cells (Fig. 4B). TMZ‐induced cell death was abrogated by CTRP8 in GBM cells, and this cytoprotective role of CTRP8 was RXFP1 dependent and blocked by RXFP1 KD with siRXFP1‐1 or siRXFP1‐2 in patient GBM‐1/2 (Fig. 4A; Figs S2E and S5E) and U87MG (Fig. 4B). Treatment with CTRP8 or siRXFP1 alone did not show cytotoxicity in patient GBM‐1/2 (Fig. 4A; Figs S2E and S5E) and U87MG (Fig. 4B). Coinciding with the cytotoxicity data, TMZ induced a significant increase in caspase 3/7 activity in patient GBM‐1/2 (Fig. 4C; Figs S2F and S5F) and U87MG (Fig. 4D). CTRP8 significantly curtailed the TMZ‐induced caspase 3/7 activation in patient GBM‐1/2 (Fig. 4C; Figs S2F and S5F) and U87MG (Fig. 4D). This anti‐apoptotic function of CTRP8 was critically dependent on the presence of a functional RXFP1‐STAT3 signaling cascade. Specific KD of RXFP1 using two different specific siRNA or treatment with STAT3 inhibitor S3I‐201 abolished this CTRP8 protective effect (Fig. 4C, D; Figs S2F and S5F). Quantitative western blot analysis demonstrated an upregulation of the anti‐apoptotic STAT3 targets Bcl‐XL and Bcl‐2 proteins in patient GBM‐1/2 (Fig. 4E, G–J; Fig. S5G–K) and U87MG (Fig. 4F, K–N). The upregulation of these Bcl members was abolished by RXFP1 KD (Fig. 4E, F, G, I, K, M; Fig. S5G, H, J) and STAT3 inhibition (Fig. 4E, F, H, J, L, N; Fig. S5G, I, K). In summary, the ability of the novel CTRP8‐RXFP1‐STAT3 signaling axis to guard against TMZ‐induced DNA damage and promote survival pathways provide evidence for a novel role of the CTRP8‐RXFP1 system in TMZ chemoresistance in human GBM (Fig. 5).

## 4 Discussion

The current gap in our understanding of the cellular mechanisms employed by human GBM to effectively thwart drug‐mediated DNA alkylating damage contributes to a lack in therapeutic improvement and the dismal prognosis of GBM patients. Here, we demonstrate a novel mechanism which links our recently discovered autocrine/paracrine CTRP8 activation of the G protein‐coupled receptor RXFP1 with the oncogenic STAT3 signaling pathway predictive of poor clinical outcome in human GBM patients (Birner *et al*., 2010; Glogowska *et al*., 2013). Our data provide first evidence that an activated CTRP8‐RXFP1‐STAT3 axis promotes BER and increases resistance to the first‐line chemotherapeutic drug TMZ in human GBM cells. Key clinical features of GBM pathology include extensive cerebral dissemination and resistance to treatment with chemotherapeutic drugs such as TMZ. CTRP8‐RXFP1 is emerging as a new ligand–receptor system which promotes GBM migration (Glogowska *et al*., 2013) and, as shown here, protects against the cytotoxic effects of the DNA alkylating drug TMZ. Likely initiated by an interaction of RXFP1 with the small G protein Gα_i3_ to activate the Gα_i3_‐Gβγ‐PI3K signaling pathway (Nguyen and Dessauer, 2005), our discovery of a novel CTRP8‐RXFP1‐STAT3 signaling cascade in human GBM links this CTRP8‐RXFP1 system to oncogenic STAT3 functional outcomes, including GBM cell survival, angiogenesis, and cell migration/invasion (Aziz *et al*., 2010; Butler *et al*., 2013; Ouedraogo *et al*., 2017). CTRP8‐activated RXFP1 may utilize PI3K to mediate STAT3 activation as PI3K and its target BMX TEC kinase were recently shown to mediate the phosphorylation of STAT3 (Glogowska *et al*., 2013; Hart *et al*., 2011). We also previously identified lysosomal cathepsins as targets of H2 relaxin, the cognate ligand of RXFP1, and CTRP8 in human thyroid cancer (cathepsin‐D and cathepsin‐L) and GBM (cathepsin‐B), respectively (Glogowska *et al*., 2013; Hombach‐Klonisch *et al*., 2006). High cathepsin‐B serum levels are associated with poor prognosis in GBM patients (Strojnik *et al*., 2005). Stat3 upregulates the expression of lysosomal proteases cathepsin‐B and cathepsin‐L under physiological conditions (Kreuzaler *et al*., 2011) and, thus, may facilitate cathepsin‐B enhanced tissue invasion and lysosomal‐mediated cell death regulation in brain tumors (Levicar *et al*., 2002).

Although TMZ is the drug of choice in the treatment of GBM patients, frequent treatment failures result in resistance to this drug and fatal GBM recurrences (Furnari *et al*., 2007; Sarkaria *et al*., 2008). Major DNA adducts generated by TMZ are N^7^‐methylguanine (N^7^‐MeG; 60‐80%), N^3^‐methyladenine (N^3^‐MeA; 10–20%), and *O* ^6^‐methylguanine (*O* ^6^‐MeG; 5–10%) (Bobola *et al*., 2012). Excision of a modified base generates an apurinic/apyrimidinic (AP) DNA site, and consecutive AP endonuclease 1 (APE1) activity creates a single‐stranded DNA site which has the propensity to develop into a double‐strand break if not processed expediently by BER (Helena *et al*., 2018). BER is the predominant DNA repair pathway for the repair of single cytotoxic DNA base lesions which includes oxidized, deaminated, and N^7^‐MeG/N^3^‐MeA alkylated nucleotides (Kim and Wilson, 2012; Krokan and Bjoras, 2013), whereas TMZ‐induced cytotoxic, radio‐sensitizing, and base‐mispairing *O* ^6^‐MeG sites are removed by the *O* ^6^‐MeG DNA methyltransferase (MGMT). MGMT is the sole enzyme dedicated to the demethylation of *O* ^6^‐meG to guanine by transferring this methyl group to an internal cysteine residue which inactivates the MGMT enzyme (Spiegl‐Kreinecker *et al*., 2010). Although MGMT promoter hypermethylation in GBM tumors is clinically associated with a better initial TMZ treatment response (Hegi *et al*., 2005), even MGMT‐negative GBM cells do not sufficiently respond to TMZ. This demonstrates the need to identify additional molecular mechanisms contributing to TMZ resistance. Our data showed that the DNA protective role of CTRP8 was as effective in MGMT‐negative U87MG and MGMT‐positive patient GBM cells (Thanasupawat *et al*., 2017a), excluding altered cellular MGMT enzyme levels as a target of activated RXFP1 and cause for the CTRP8‐mediated protection against TMZ‐induced DNA damage in our GBM models. We therefore focused on N^7^‐MeG and N^3^‐MeA adducts which constitute over 90% of TMZ‐induced base alterations. These are recognized by the BER glycosylase MPG which performs the initial cleavage of the glycosylic bond between the damaged base and deoxyribose to generate an AP site (Bobola *et al*., 2012; Kim and Wilson, 2012). Treatment of human GBM cells with RXFP1 agonist CTRP8 increased MPG protein levels and MPG activity as determined by MPG molecular beacon assay, while the cellular levels of other BER proteins, including APE1, DNA polymerase β (polβ), and XRCC1, remained unaffected by CTRP8‐mediated RXFP1 activation. The increased MPG activity resulted in enhanced BER capacity, reflected by the reduced number of AP sites and DNA double‐strand breaks with resulting decrease in apoptosis. This indicated sufficient activity of BER factors downstream of MPG to ensure enhanced BER capacity in GBM with activated RXFP1 (Trivedi *et al*., 2008).

TMZ resistance in GBM is associated with DNA damage‐induced activation of the serine/threonine ataxia telangiectasia mutated kinase (ATM). Phosphorylation of MPG by ATM coincides with increased MPG activity and has been linked to alkylating drug resistance in pediatric GBM (Agnihotri *et al*., 2014). However, it should be noted that this kinase‐mediated mechanism of MPG activation utilizes preexisting cellular MPG as phosphorylation substrate, likely as a fast response to DNA damage. By contrast, the activation of the CTRP8‐RXFP1‐STAT3 signaling cascade initiated MPG gene activation and increased MPG protein production, consequently resulting in higher and possibly more sustained MPG glycosylase activity in stressed GBM. MPG has been described as an unfavorable independent prognostic factor for glioma patients and MPG gene and protein expression increase from low‐ to high‐grade gliomas (Liu *et al*., 2012). Glioma patients undergoing TMZ treatment with low MPG levels, possibly due to MPG promoter methylation, have a better outcome compared to those with high MPG expression (Agnihotri *et al*., 2012). This points to the importance of a proper balance of BER factors and links increased MPG activity and AP site formation to enhanced TMZ resistance in GBM (Tang *et al*., 2011). Recently, the drug salinomycin was shown to downregulate the expression of DNA repair factors MPG, MGMT, and Rad51 recombinase and induce endoplasmic reticulum (ER) stress. Combined salinomycin/TMZ treatment of GBM cells resulted in enhanced TMZ sensitivity, DNA damage, apoptosis, and increased survival of mice with orthotopic GBM xenografts (Xipell *et al*., 2016). The ability to enhance TMZ resistance and mount an anti‐apoptotic Bcl2‐like response identifies CTRP8‐RXFP1 as a new and powerful defense against TMZ stress in glioma. All the results presented here for CTRP8 were replicated with H2 relaxin in our human GBM models and yielded similar results (T. Klonisch, personal communication). While this confirmed a key role for GBM‐expressed CTRP8 as oncogenic driver in human glioblastoma, it is conceivable that H2 relaxin has a similar effect on therapeutic resistance in RXFP1+ tumors outside of the brain. The Rembrandt database identifies RXFP1 as constitutively expressed gene in all human GBM, suggesting subtype independent roles of RXFP1 in GBM.

## 5 Conclusions

Ligand binding to the G protein‐coupled receptor RXFP1 and downstream STAT3 pathway activation protects GBM against the DNA alkylating drug temozolomide. This TMZ resistance is mediated by STAT3 upregulation of MPG glycosylase with enhanced BER and increases Bcl‐2/Bcl‐XL anti‐apoptotic pathway activation in human GBM cells. Thus, the RXFP1 ligand–receptor system should be considered an attractive new drug target to curb TMZ resistance in GBM.

## Author contributions

TT, AG, and MB performed the experimental work. JK, JB, and MP assisted in data collection and data analysis. GJZ provided critical revisions of the manuscript. SHK and TK conceived the study. TK lead the study and drafted the manuscript. All authors approved of the final version of this manuscript prior to submission.

## Supporting information


**Fig. S1.** CTRP8 promotes STAT3 signaling in another patient GBM‐2 cell model.
**Fig. S2.** Different siRNA confirms essential role of RXFP1 in CTRP8 function.
**Fig. S3.** CTRP8 attenuates TMZ induced DNA damage in different human GBM models.
**Fig. S4.** CTRP8 diminishes dsDNA breaks in the patient GBM‐2 model.
**Fig. S5.** CTRP8 enhances MPG and promotes survival in the second patient GBM model.
**Fig. S6.** *O* ^6^‐methylguanine DNA methyltransferase (MGMT) is not a target of CTRP8.
