# Supplementary material for: C1q/TNF‐related peptide 8 (CTRP8) promotes temozolomide resistance in human glioblastoma
Source: Mol Oncol. 2018 Aug 2;12(9):1464–79. doi: 10.1002/1878-0261.12349 (PMC6120254; doi:10.1002/1878-0261.12349)
Supplement: Supplementary file 1 — Fig. S1. CTRP8 promotes STAT3 signaling in another patient GBM‐2 cell model. Fig. S2. Different siRNA confirms essential role of RXFP1 in CTRP8 function. Fig. S3. CTRP8 attenuates TMZ induced DNA damage in different human GBM models. Fig. S4. CTRP8 diminishes dsDNA breaks in the patient GBM‐2 model. Fig. S5. CTRP8 enhances MPG and promotes survival in the second patient GBM model. Fig. S6. O 6‐methylguanine DNA methyltransferase (MGMT) is not a target of CTRP8. [file MOL2-12-1464-s001.pdf]

Suppl. Figure 1.

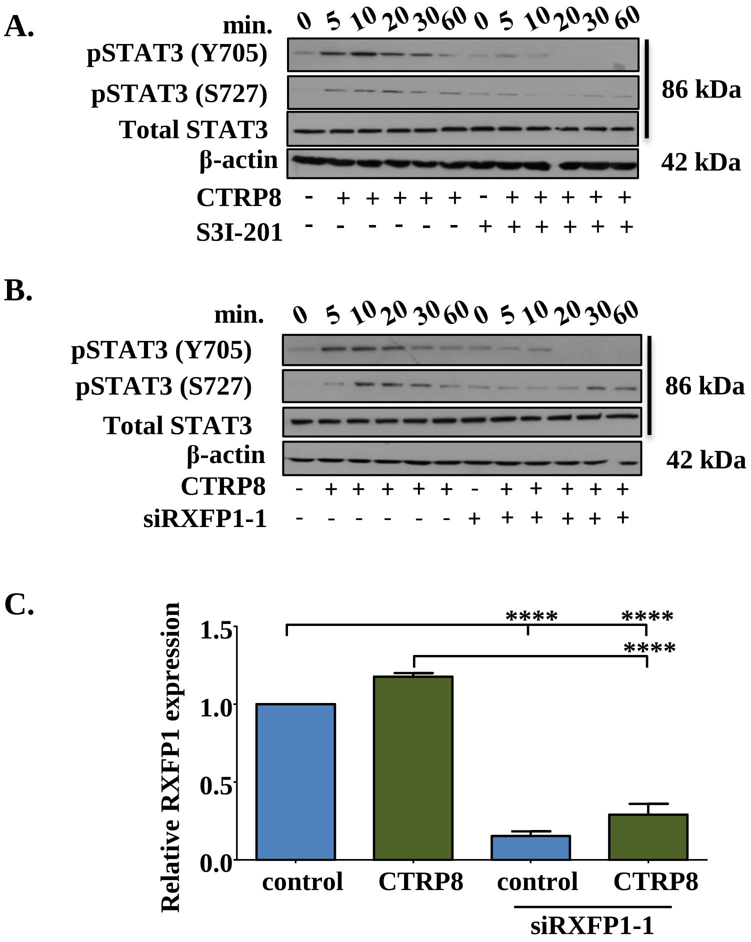

Supplemental Figure 1. CTRP8 promotes STAT3 signaling in another patient GBM-2 cell model. Exposure of CTRP8 resulted in the phosphorylation of STAT3 at Tyr^{705} and Ser^{727} (**A**). Pretreatment with the specific STAT3 inhibitor S3I-201 abolished the ability of CTRP8 to cause STAT3 phosphorylation (**A**). The ability of CTRP8 to promote the phosphorylation of both $pSTAT3^{Y705/S727}$ residues was dependent on RXFP1 diminished upon siRXFP1-1 KD (**B**). β-actin served as loading control. Upon siRXFP1-1 treatment, qPCR demonstrated a significant downregulation of RXFP1 transcripts (**C**). Quantitative analysis from three independent experiments (two-way ANOVA; data are shown as mean ± SD; ****$p<0.0001$) are shown.

Suppl. Figure 2.

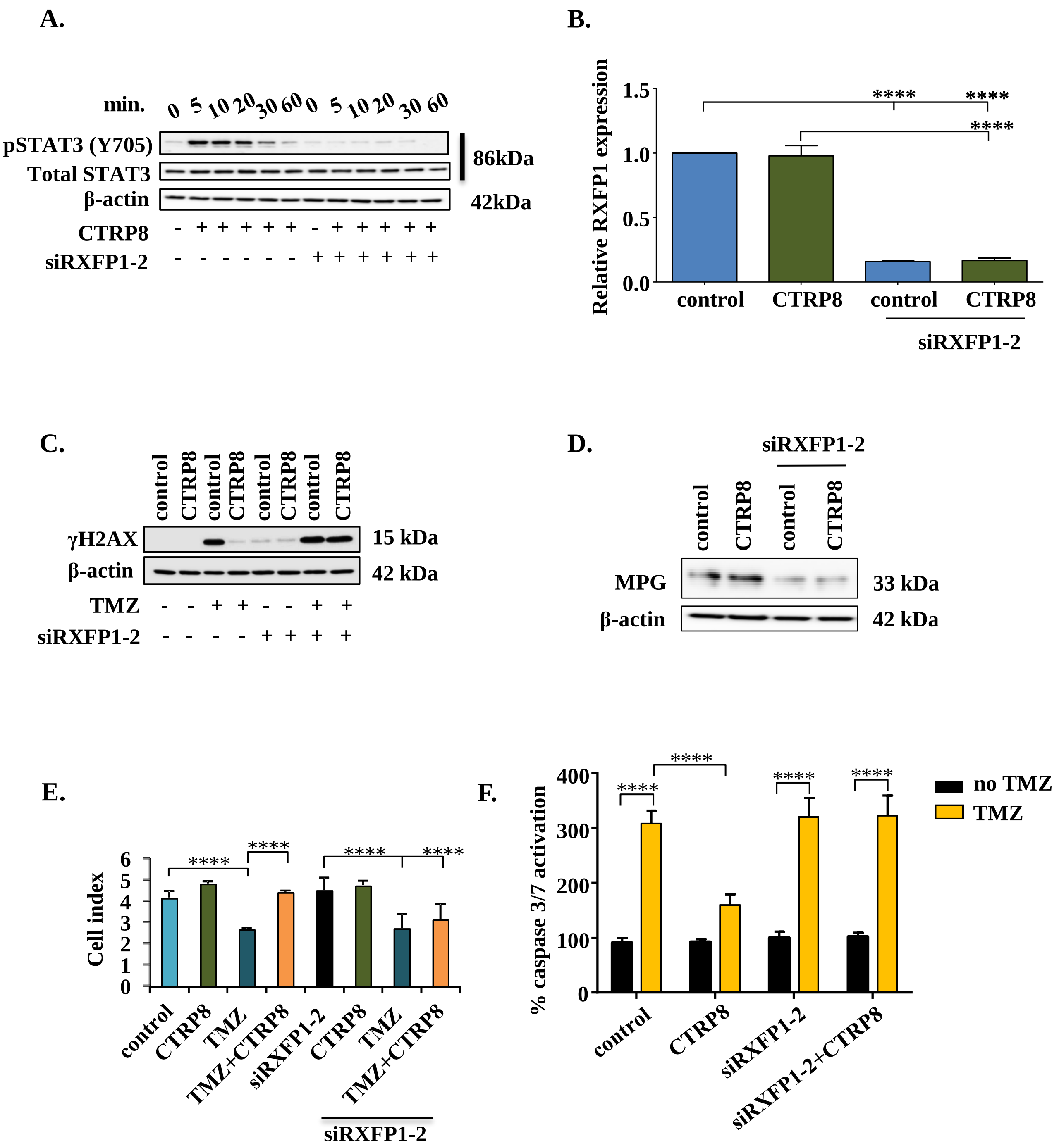

Supplemental Figure 2. Different siRNA confirms essential role of RXFP1 in CTRP8 function. To exclude siRNA induced off-target effects, we performed key experiments using a different siRNA to achieve RXFP1 KD in patient GBM-2 cells. Western blot analysis showed increased phosphorylation of $STAT3^{Tyr705}$ upon treatment with CTRP8. RXFP1 KD with the second siRXFP1-2 abolished the ability of CTRP8 to cause $STAT3^{Tyr705}$ phosphorylation (**A**). QPCR demonstrated successful RXFP1 KD upon treatment with the second siRXFP1-2 (**B**). Upon TMZ treatment, levels of γH2AX significantly decreased in the presence of CTRP8 and this reduction in γH2AX was inhibited upon treatment with siRXFP1 (**C**). The CTRP8 induced upregulation of MPG glycosylase protein expression was abolished upon treatment with siRXFP1-2 (**D**). β-actin served as loading control. Real-time xCelligence assays demonstrated that CTRP8 diminished the toxicity of TMZ on patient GBM-2 cells (**E**). However, this protective CTRP8 effect was abolished upon treatment with the second set of siRXFP1-2 (**E**). TMZ cytotoxicity coincided with increased caspase3/7 activation in patient GBM-2 cells which was reduced in the presence of CTRP8. Similar to our results with the first set of siRXFP1-1 (*Fig. 4C*), this cytoprotective effect of CTRP8 depended on RXFP1 and was lost upon treatment with siRXFP1-2 (**F**). Hence, the effects of CTRP8 on human glioblastoma required the presence of RXFP1 and a functional STAT3 signaling cascade and were not the result of siRNA off-target effects. Quantitative analysis from three independent experiments (two-way ANOVA; data are shown as mean ± SD; $****p<0.0001$) are shown.

Suppl. Figure 3.

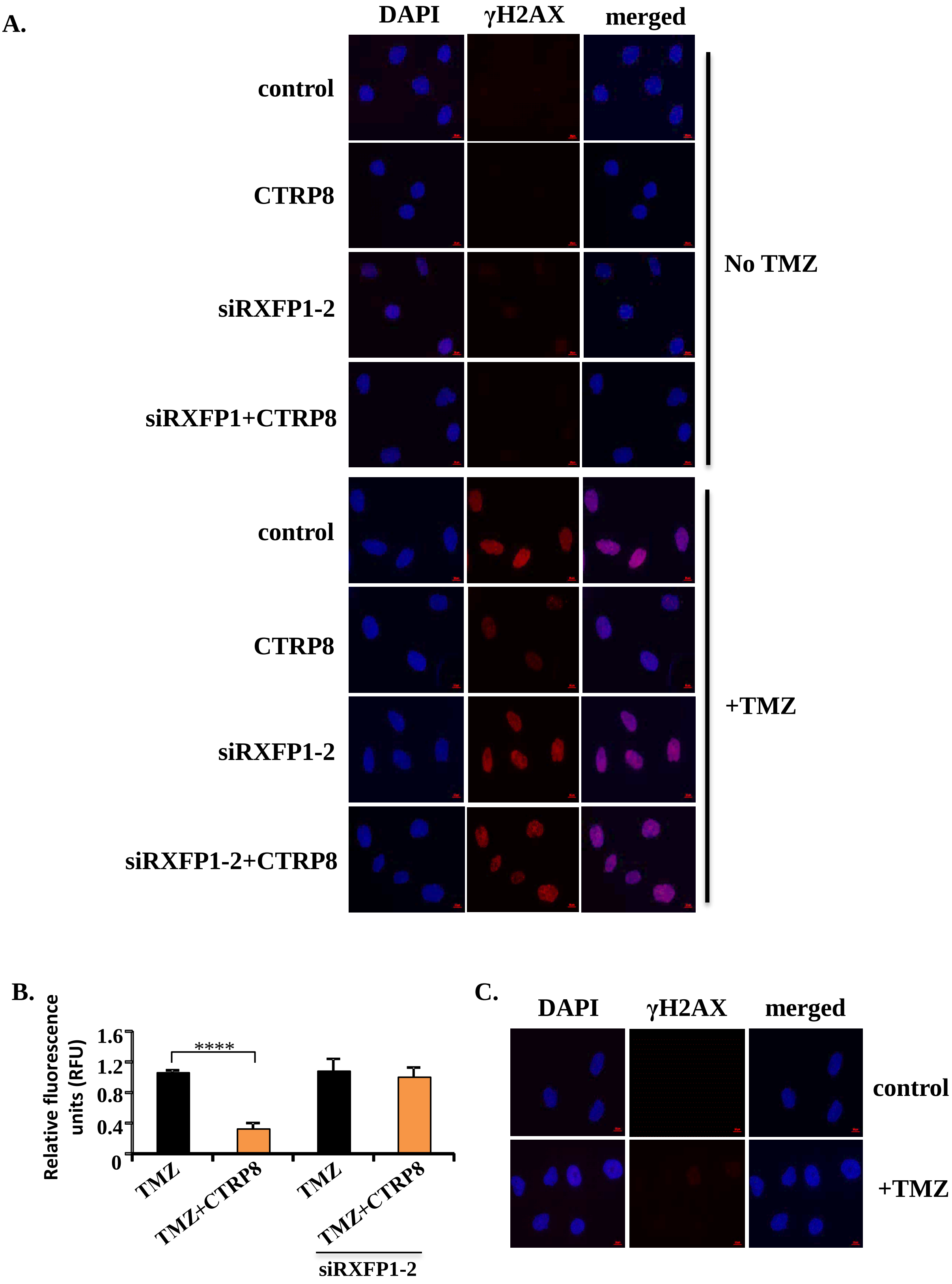

Suppl. Figure 3.

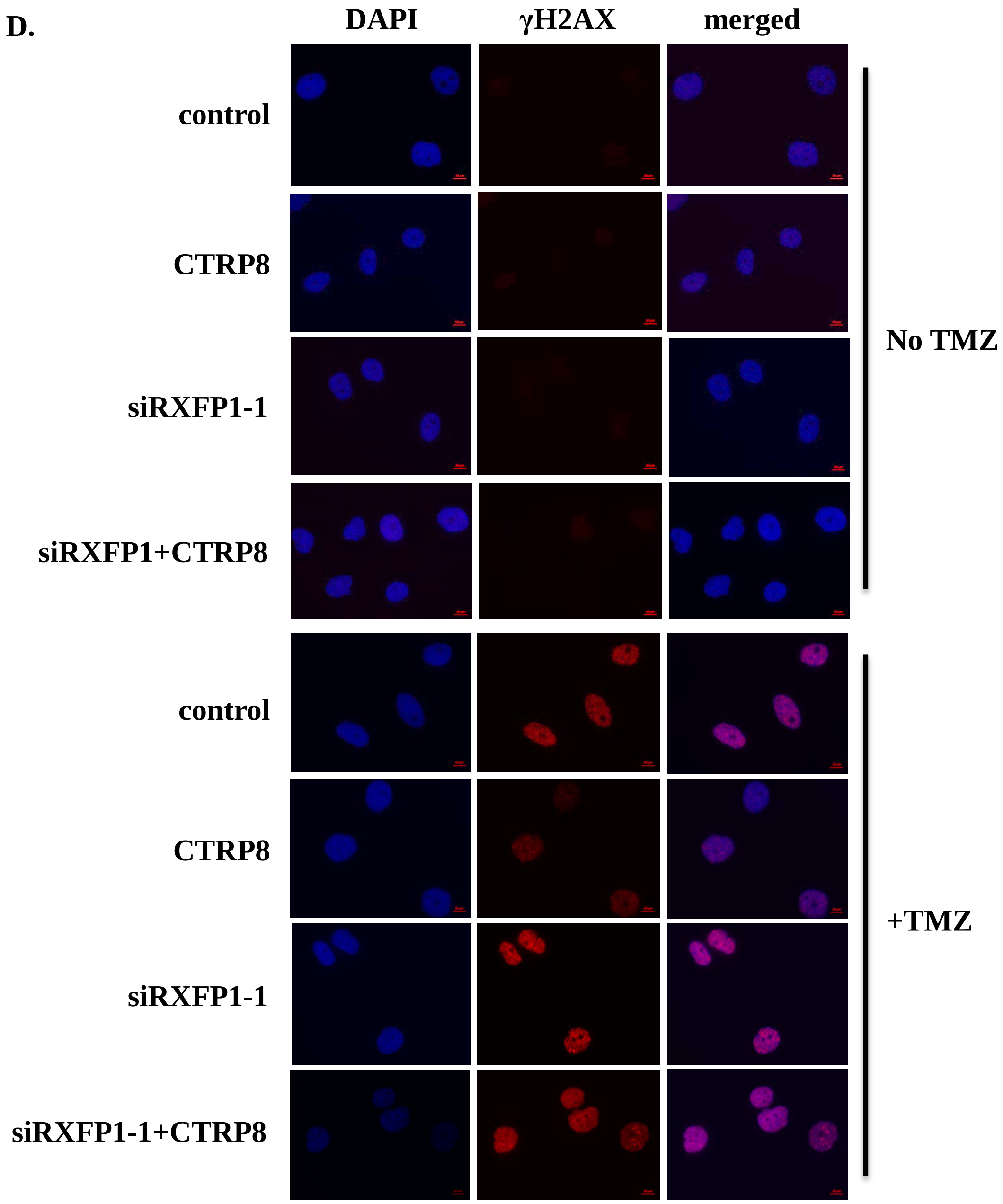

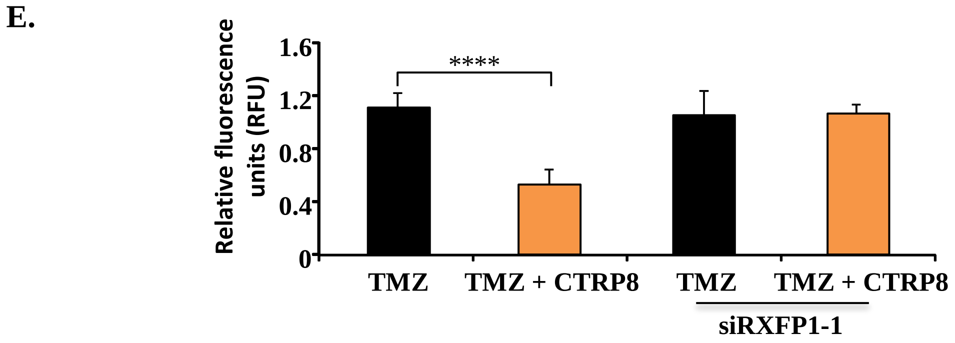

Suppl. Figure 3.

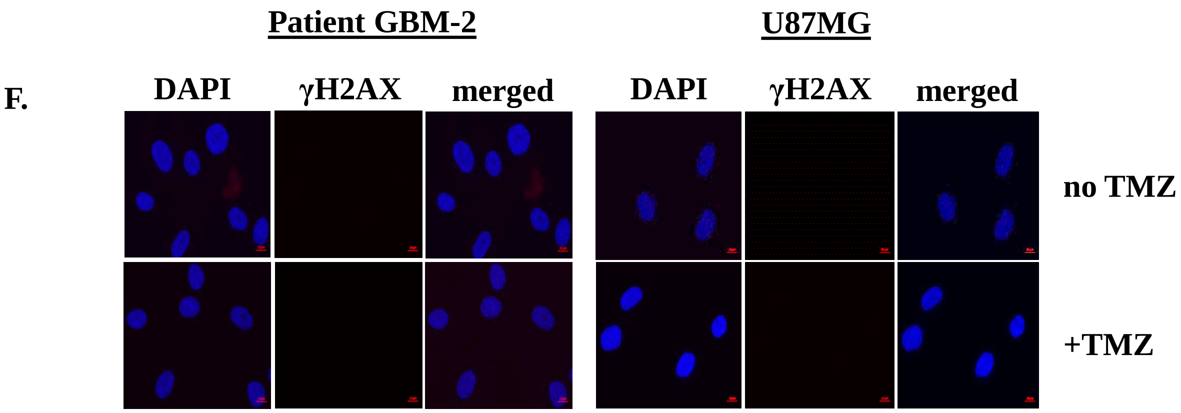

Supplemental Figure 3. CTRP8 attenuates TMZ induced DNA damage in different human GBM models. Quantitative immunofluorescence performed on GBM cells from a different GBM patient (GBM-2) (**A-C**) and U87MG (**D-F**) demonstrated a significant increase in γH2AX fluorescence intensity upon TMZ treatment which was diminished in the presence of CTRP8 (**A, B, D, E**). This CTRP8 mediated DNA protective effect was blocked by siRXFP1-1/2 KD (**A, B, D, E**). Quantification of γH2AX fluorescence intensity was done in 100 nuclei for each treatment (**B, E**). Mouse IgG isotype controls showed no specific staining and served as negative controls for all GBM cell models (**C, F**). Quantitative analysis from three independent experiments (two-way ANOVA; data are shown as mean ± SD; ****$p<0.0001$) are shown.

Suppl. Figure 4.

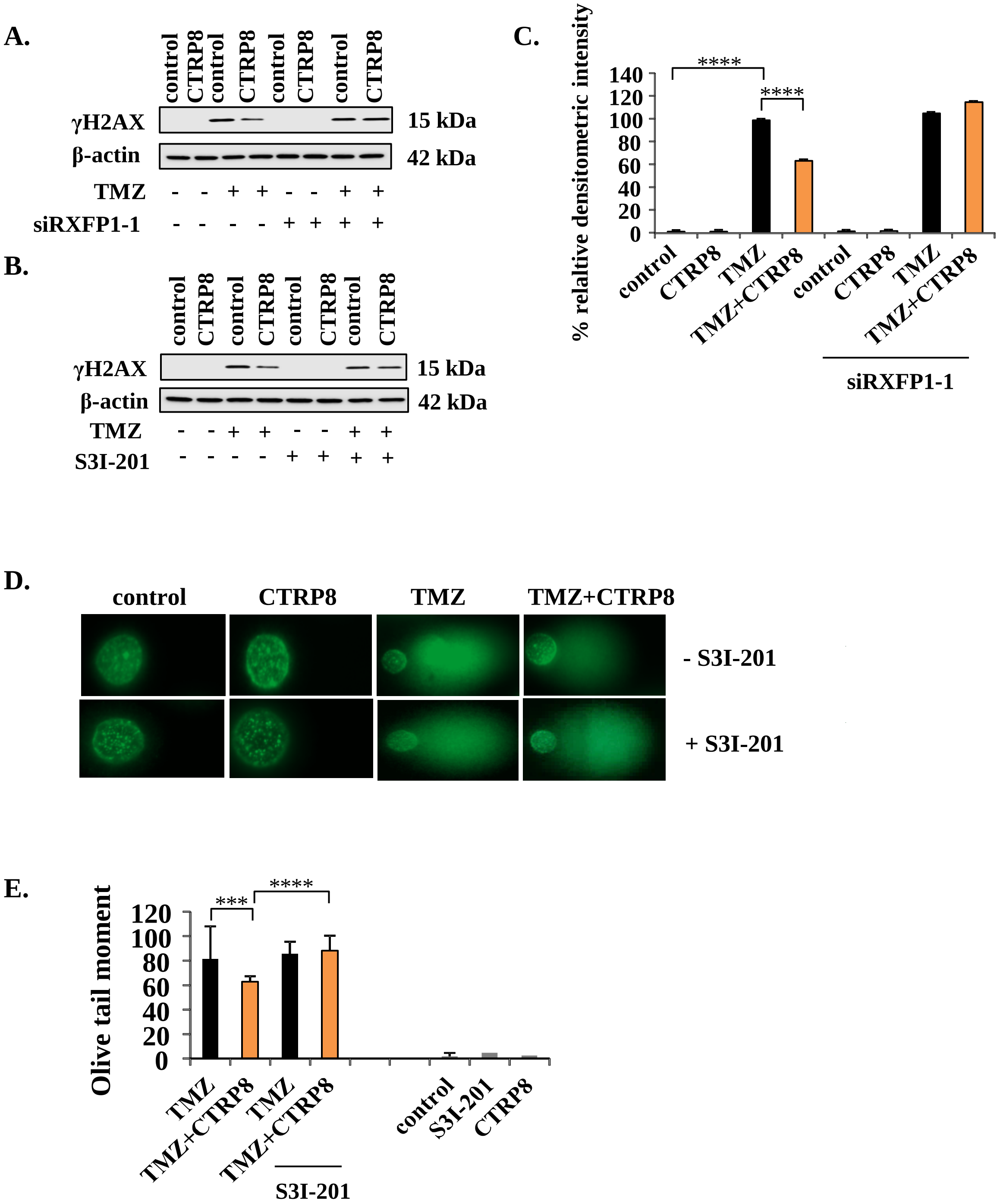

Supplemental Figure 4. CTRP8 diminishes dsDNA breaks in the patient GBM-2 model. Representative quantitative Western blot analysis of γH2AX revealed an increase in γH2AX fluorescence signals in TMZ treated patient GBM-2 cells which was significantly diminished in the presence of CTRP8 (**A-C**). This DNA protective function of CTRP8 was dependent on the presence of functional RXFP1-STAT3 signaling and abolished upon treatment with siRXFP1-1 (**A, C**) or STAT3 inhibitor S3I-201 (**B**). Quantitative single cell DNA damage analysis (Comet assays) performed on the second patient GBM model GBM-2 revealed similar results to those obtained with patient GBM-1 (*Fig. 2I-M*). TMZ treatment increased the olive tail moment (OTM) in these patient GBM-2 cells, indicating frequent double strand DNA breaks (**D, E**). This TMZ induced DNA damage was diminished by CTRP8 treatment (**D**). Quantitative analysis of OTM in 50 cells for each treatment revealed that CTRP8 caused a marked reduction in dsDNA breaks (**E**) but this DNA protective CTRP8 function was lost upon treatment with S3I-201 (**E**). CTRP8 and S3I-201 alone failed to cause DNA breaks and resulted in negligible OTM (**E**). Quantitative analysis from three independent experiments (two-way ANOVA; data are shown as mean ± SD; ***$p<0.001$, and ****$p<0.0001$) are shown.

Suppl. Figure 5.

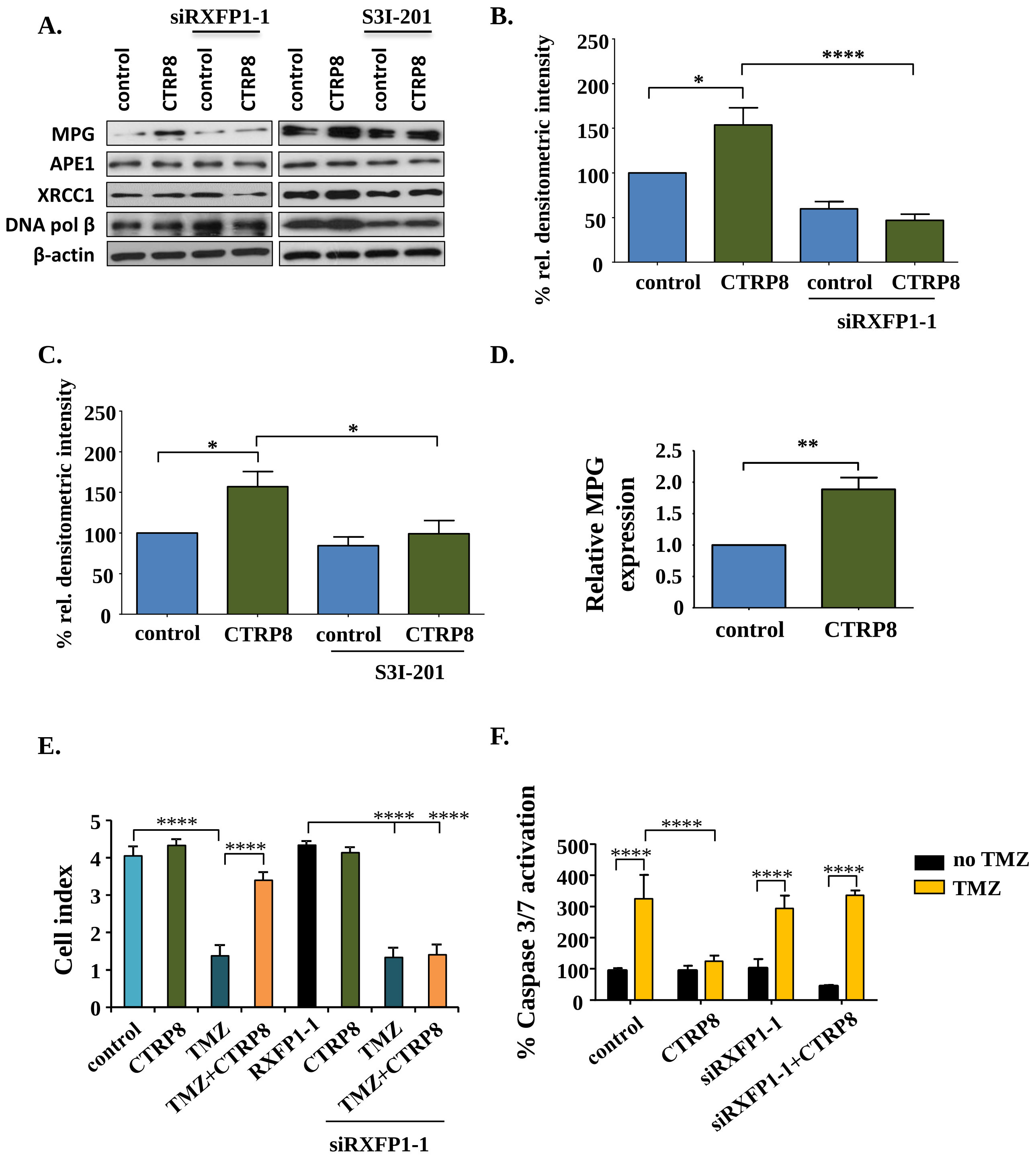

Suppl. Figure 5.

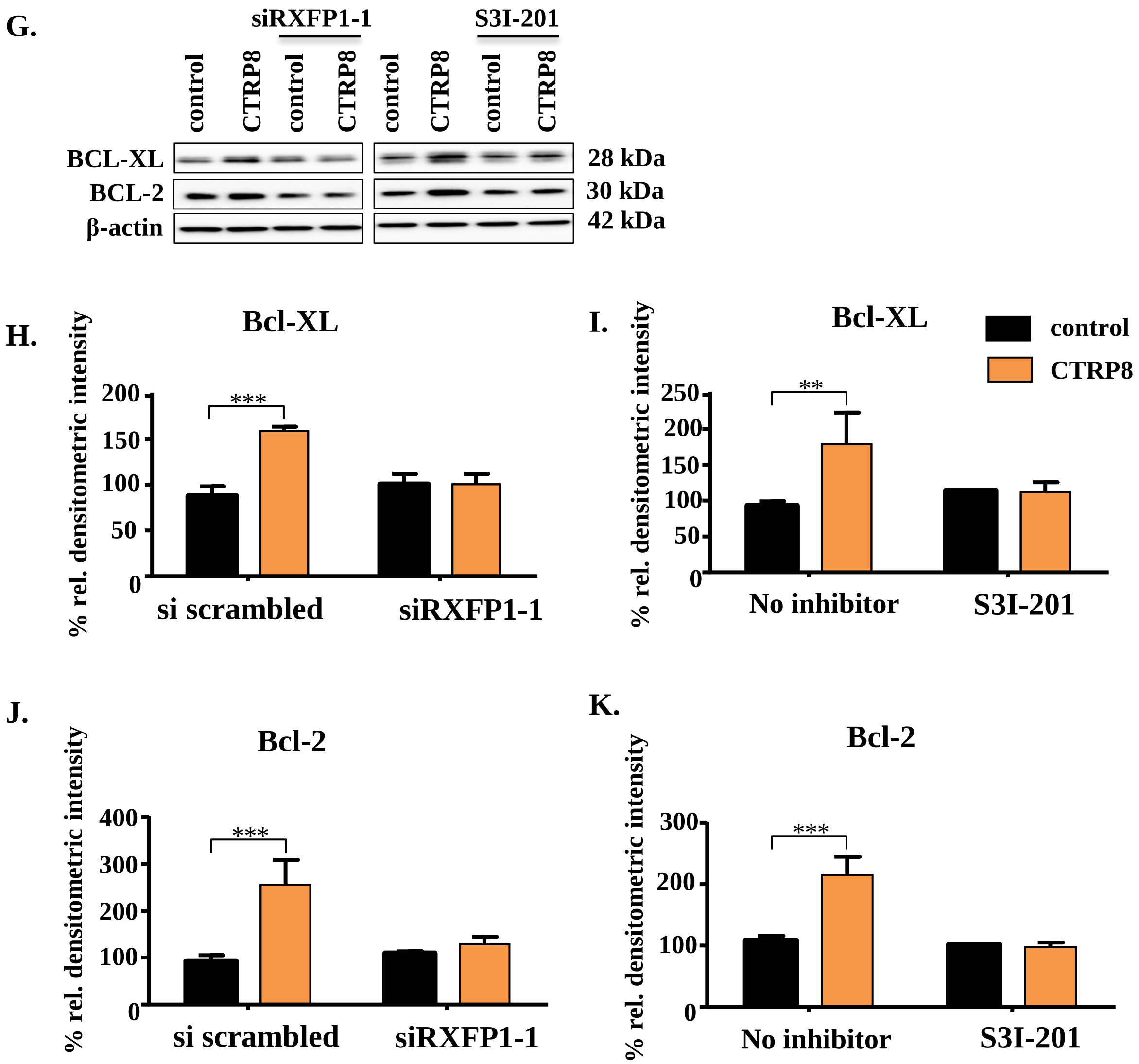

Supplemental Figure 5. CTRP8 enhances MPG and promotes survival in the second patient GBM model. Representative Western blots (**A**) and corresponding quantitative analysis (**B, C**) done on GBM-2 cells showed that CTRP8 treatment resulted in a marked increase in DNA glycosylase MPG protein (**A**). SiRXFP1 KD (**B**) or STAT3 inhibition (**C**) abolished this increase in MPG protein by CTRP8 (**A-C**), indicating the requirement for a functional RXFP1-STAT3 signaling cascade. The upregulation of MPG protein coincided with a significant increase in MPG transcripts as determined by qPCR (**D**). TMZ cytotoxicity was diminished by CTRP8 in GBM-2 as determined by real-time xCelligence assays (**E**). This protective effect of CTRP8 was dependent on RXFP1 and abolished by siRXFP1-1 (**E**). The presence of RXFP1 was also required for the suppressive effect of CTRP8 on caspase3/7 activation upon TMZ treatment (**F**). Quantitative Western blot analysis showed that a CTRP8 mediated upregulation of Bcl-XL and Bcl-2 proteins (**G**). The upregulation of both anti-apoptotic Bcl members was dependent on the presence of a functional RXFP1-STAT3 signaling pathway (**H-K**). Collectively, our results obtained with this second patient GBM cell model (GBM-2) were in agreement with those of GBM-1 investigated (*Fig. 3B-I; Fig. 4A-N*). Quantitative analysis from three independent experiments (two-way ANOVA; data are shown as mean ± SD; *$p<0.05$, **$p<0.01$, ***$p<0.001$, and ****$p<0.0001$) are shown.

Suppl. Figure 6.

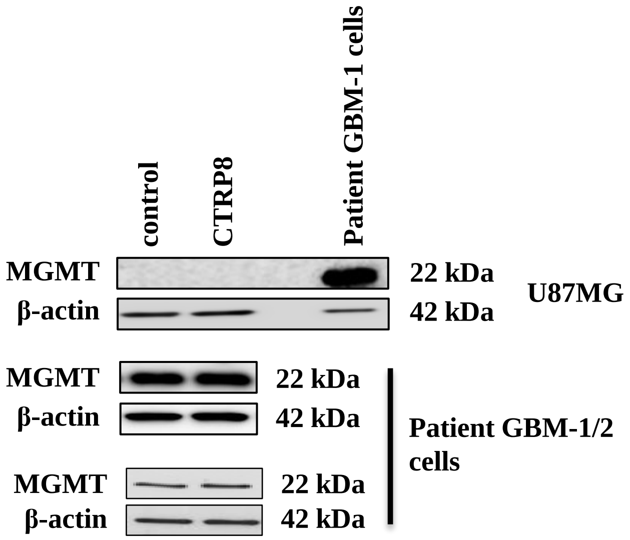

Supplemental Figure 6. O^6-methylguanine DNA methyltransferase (MGMT) is not a target of CTRP8. MGMT protein expression was determined by Western blots in both patient GBM-1/2 models studied. U87MG was negative for MGMT. The two patient GB models expressed MGMT but cellular protein levels remained unchanged upon CTRP8 treatment, indicating that this DNA repair protein is not a target of CTRP8.
